# Use of [^18^F]Fluorodeoxyglucose Positron Emission Tomography/Computed Tomography after Curative Treatment of Non-Small-Cell Lung Cancer Patients: A Nationwide Cohort Study

**DOI:** 10.3390/diagnostics14020233

**Published:** 2024-01-22

**Authors:** Kasper Foged Guldbrandsen, Liza Sopina, Torben Riis Rasmussen, Barbara Malene Fischer

**Affiliations:** 1 Department of Clinical Physiology and Nuclear Medicine, Copenhagen University Hospital-Rigshospitalet, 2100 Copenhagen, Denmark; 2 Danish Center for Health Economics (DaCHE), University of Southern Denmark (SDU), 5230 Odense, Denmark; 3 Department of Respiratory Diseases and Allergy, Aarhus University Hospital, 8200 Aarhus, Denmark

**Keywords:** non-small-cell lung cancer, follow-up, FDG PET, healthcare utilization

## Abstract

[¹⁸F]Fluorodeoxyglucose positron emission tomography/computed tomography ([¹⁸F]FDG PET/CT) is a valuable imaging tool in the post-treatment management of non-small-cell lung cancer (NSCLC). This study aimed to investigate the trends in utilization and factors associated with the use of [¹⁸F]FDG PET/CT after curative-intent treatment. Data from 13,758 NSCLC patients diagnosed between 2007 and 2020 identified in the Danish Lung Cancer Registry, who underwent curative-intent treatment, were analyzed using multivariable regression. The results showed a significant increase in the use of [¹⁸F]FDG PET/CT scans, from 10.4 per 100 patients per year in 2007 to 39.6 in 2013, followed by a period of stability. Higher utilization rates were observed in patients who received radiotherapy (22% increase compared to surgical resection) and in patients with stage II–III disease (14% and 20% increase compared to stage I, respectively). Additionally, utilization was increased when other diagnostic procedures were performed, such as MRI, ultrasound, endoscopy, and biopsy. These findings highlight an increasing reliance on [¹⁸F]FDG PET/CT in post-treatment NSCLC, especially after radiotherapy and in patients with locally advanced disease, where treatment-induced radiographic changes and an increased risk of recurrence present a significant diagnostic challenge.

## 1. Introduction

Lung cancer carries a poor prognosis, even for patients diagnosed at an early stage and eligible for curative-intent treatment [1]. The risk of recurrence is as high as 40% in the first years following curative-intent treatment, depending on the initial stage and treatment type [2]. Consequently, guidelines recommend close surveillance after therapy to detect recurrence. Nonetheless, the optimal frequency and type of imaging are still contentious subjects due to a lack of high-quality prospective studies [3].

Most guidelines agree that a CT scan should be performed every six months for the first two to three years following therapy and then annually [4]. Nonetheless, despite routine surveillance, one-third of recurrences are detected outside scheduled surveillance, and most patients who experience recurrence have distant metastasis at the time of diagnosis, which limits the available treatment options [5]. These findings emphasize the need for improved surveillance techniques with greater sensitivity and specificity for detecting recurrences, which could help detect recurrences earlier and potentially improve patient outcomes.

[¹⁸F]Fluorodeoxyglucose positron emission tomography/computed tomography ([¹⁸F]FDG PET/CT) has, in recent years, become an invaluable tool in managing patients with lung cancer. One of the most successful applications of this technology in lung cancer has been the adoption of [¹⁸F]FDG PET/CT for preoperative staging. Compared to conventional staging methods, [¹⁸F]FDG PET/CT improves the detection of mediastinal and distant metastasis, establishing it as an essential tool for determining which patients would benefit from surgical resection [6]. Further, [¹⁸F]FDG PET/CT has an excellent diagnostic performance for recurrence detection after curative-intent therapy, especially in patients with a high risk of recurrence [7]. In addition, [¹⁸F]FDG PET/CT may benefit a subset of patients with suspicious findings that are difficult to characterize on CT during post-treatment surveillance [8]. Due to this, [¹⁸F]FDG PET/CT is recommended for evaluating suspected recurrence, but it is not recommended to be used as a surveillance tool in patients without any suspicion of recurrence [9]. However, little is known about how [¹⁸F]FDG PET/CT is currently utilized in lung cancer patients during the surveillance period following curative-intent therapy.

To better understand the evolving role of [¹⁸F]FDG PET/CT in the management of lung cancer patients after treatment, we conducted a retrospective analysis based on a national cohort of patients with NSCLC treated with curative intent. This study aims to investigate how the use of [¹⁸F]FDG PET/CT during post-treatment surveillance has changed over time, especially since 2007, when [¹⁸F]FDG PET/CT started gaining widespread use for lung cancer staging, and to identify the demographic and clinical factors associated with the use of [¹⁸F]FDG PET/CT during post-treatment surveillance.

## 2. Materials and Methods

### 2.1. Data Source and Study Population

This retrospective study was conducted using data from the Danish Lung Cancer Registry (DLCR). The DLCR is a national longitudinal database that contains clinical and demographic data on all patients diagnosed with lung cancer in Denmark since 2000 [10]. The DLCR comprises data from the Danish National Patient Registry (NPR), the Danish Pathology Register, and the Danish Civil Registration System (CPR). Clinicians involved in diagnosing or treating patients with lung cancer supplement and validate the data in the DLCR. The registry provides comprehensive information on patient demographics, cancer staging, treatment, and outcomes. The DLCR also provides data on all lung cancer-related procedures performed on patients in its database using the Danish Medical Coding Classification System (DMCCS). From the DLCR, we identified all patients diagnosed with stage IA-IIIC NSCLC from 1 January 2007 to 31 December 2020. The extracted variables for each patient included patient age, sex, date of diagnosis, region of residence, clinical and pathological stage, vital status, and treatment received. Additionally, we extracted all the procedure data of each patient, which were available up to 1 June 2021.

From 2019 to 2021, a national randomized controlled trial recruited patients with NSCLC in Denmark to compare CT scans and [¹⁸F]FDG PET/CT as a surveillance tool after curative treatment [11]. Because imaging use in patients enrolled in this trial is expected to differ from standard follow-up care after curative treatment, patient inclusion status in the trial was also considered in this analysis.

### 2.2. Procedures

For each patient, we identified post-treatment imaging and interventions performed during a two-year period after curative therapy. The start of the two years was set to either three months after surgical resection or six months after the start of curative-intent radiotherapy, depending on what type of treatment the patient received, to minimize the inclusion of procedures related to treatment. Similarly, procedures performed three months before death were excluded from the analysis to avoid including procedures related to end-of-life care. All procedures were grouped into three diagnostic and three interventional procedure categories based on the DMCCS codes (see Table S1, DMCCS Codes). The diagnostic categories were: [¹⁸F]FDG PET/CT, CT and “other diagnostic procedures” (meaning endoscopy, biopsy, ultrasound, or magnetic resonance imaging). The interventional categories were systemic therapy (including all types of chemotherapy, targeted therapies, and immune checkpoint inhibitors), surgical resection (segmentectomy, lobectomy, or pneumonectomy), and radiotherapy (including all methods of external beam radiotherapy). Finally, we counted the number of each type of procedures performed during the two-year period using one-month intervals.

### 2.3. Exclusion Criteria

Patients who did not receive curative therapy were excluded from the analysis. Curative therapy was defined as surgical resection (with or without adjuvant chemotherapy), curative-intent stereotactic ablative radiotherapy (SABR), or definitive chemoradiation. In addition, patients with insufficient follow-up time, defined as less than three months from the start of the two-year period after therapy, were also excluded.

### 2.4. Statistical Methods

Patient characteristics are reported by year of diagnosis. Categorical variables are presented as counts and percentages, and continuous variables are presented with means and standard deviations.

A multivariate Poisson regression model based on generalized estimating equations was specified to explore whether the use of [¹⁸F]FDG PET/CT after curative treatment has changed over time and determine which factors are associated with the use of [¹⁸F]FDG PET/CT. Poisson regression was chosen to account for the count distribution of the primary outcome, which was the number of [¹⁸F]FDG PET/CT procedures performed in each 1-month interval. Generalized estimating equations were used to adjust for the correlation between repeated observations in longitudinal data for each patient [12]. Quasi-likelihood under the independence model criterion (QIC) was used to compare model performance. Based on the lowest QIC, the log link function and exchangeable correlation structure were selected for the model. Backwards selection was used to eliminate covariates that were not significant predictors of the outcome (*p* > 0.05). Missing values were imputed using multiple imputation. However, none of the significant predictors contained missing values.

The following predictors were selected for the final model: year (calendar year on the first day of each 1-month interval), “month” (from 1 to 24, with 1 representing the first 1-month interval of the two-year period and 24 representing the last), age, region of residence (i.e., which of the five healthcare regions of Denmark the patient was living in at the time of diagnosis), clinical stage (or pathological stage if available), initial treatment (either surgical resection or curative-intent radiotherapy), and inclusion in the SUPE_R trial. Additionally, the model included the following post-treatment interventions and diagnostic procedures: surgical resection, radiotherapy, systemic therapy, and “other diagnostic procedures”. Each procedure category was coded as “1” if any procedure from the group was performed at least once during a 1-month interval and “0” if none were performed. To explore differences in the use of [¹⁸F]FDG PET/CT after therapy based on the type of initial treatment, an additional model incorporating an interaction between “month” and initial treatment was used.

The results of the Poisson regression are presented as percentage change compared to the reference, as well as the adjusted utilization rate of [¹⁸F]FDG PET/CT imaging for each predictor variable. The adjusted utilization rate of [¹⁸F]FDG PET/CT imaging was estimated using predictive margins and reported as the number of [¹⁸F]FDG PET/CT procedures performed per 100 patients per year [13]. Predictive margins represent the average model predictions for the utilization rate of [¹⁸F]FDG PET/CT imaging when changing one predictor variable to a specified value and holding all other predictors constant. This method allows for estimating the adjusted frequency of [¹⁸F]FDG PET/CT imaging for each predictor variable based on the covariate distribution present in the population.

All statistical analyses were performed using R version 4.2.0, with packages geepack and marginaleffects [14,15].

## 3. Results

### 3.1. Patient Characteristics and Procedures

A total of 24,123 patients diagnosed with stage IA-IIIC NSCLC from 2007 to 2020 were identified in the DLCR. Of these, 7915 patients who did not receive curative therapy and 2450 patients with insufficient follow-up time were excluded from the analysis. A notable increase in the number of patients who received curative treatment during the study period was observed.

**Figure 1:**
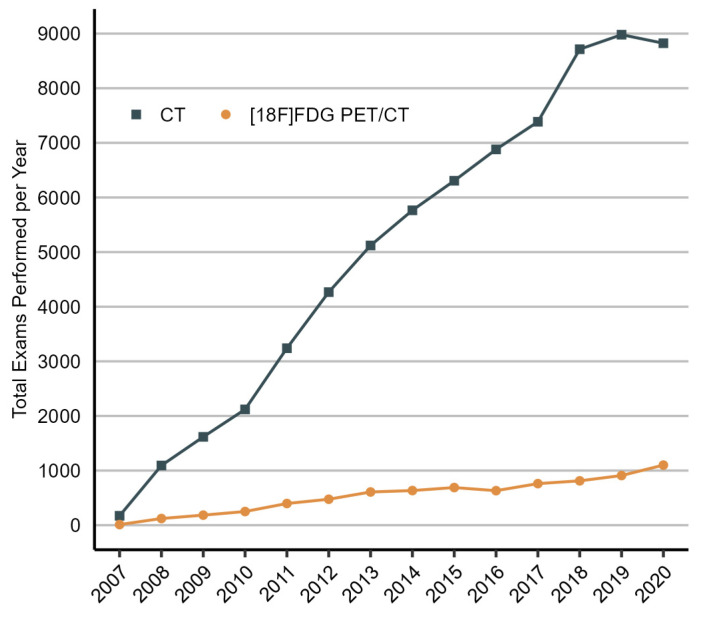
Total number of CT and [¹⁸F]FDG PET/CT scans performed in patients with NSCLC after curative treatment each year, 2007 to 2020. The increase in the number of procedures performed in the initial years of the study (2007–2009) can, in part, be attributed to the gradual inclusion of patients in the population starting from 2007. CT, computed tomography; 18F-FDG PET/CT, [¹⁸F]fluorodeoxyglucose positron emission tomography/computed tomography.

In total, 13,758 NSCLC patients who received curative treatment were included in the analysis, representing all patients with adequate follow-up undergoing such treatment in Denmark during the study period. The demographic and clinical characteristics of the included patients by year of diagnosis are presented in Table 1. The proportion of patients receiving definitive chemoradiation or SABR as the initial curative treatment increased from 21% in 2007–2008 to 32% in 2019–2020, while the number of patients undergoing surgical resection declined from 79% in 2007–2008 to 68% in 2019–2020. The proportion of patients who received at least one [¹⁸F]FDG PET/CT scan during the two-year post-treatment period increased from 23% in 2007–2008 to 43% in 2017–2018, and the proportion of patients who received at least one CT scan increased from 77% in 2007–2008 to 98% in 2019–2020. The reduction in the number of patients who received at least one[¹⁸F]FDG PET/CT scan, from 43% in 2017–2018 to 38% in 2019–2020, is likely due to the decreased follow-up time of patients diagnosed in 2019–2020. From 2008 to 2020, the number of [¹⁸F]FDG PET/CT procedures performed in the population grew by 18% each year, and the number of CT procedures increased by 17% (Figure 1). Post-treatment interventions were performed in 34% of patients.

### 3.2. Multivariate Analysis

**Figure 2:**
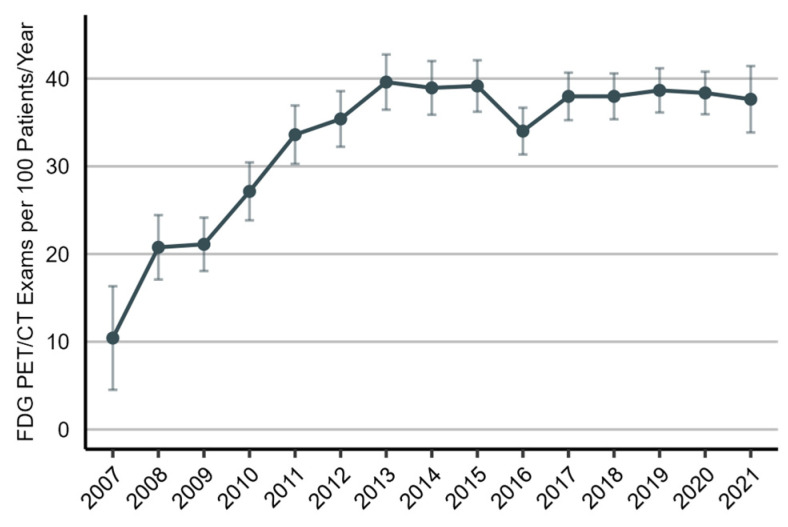
Utilization rate of [¹⁸F]FDG PET/CT imaging in the post-treatment management of non-small-cell lung cancer patients, 2007 to 2021. The utilization rate is presented as the number of [¹⁸F]FDG PET/CT scans per 100 patients per year (predictive margins), accompanied by a 95% confidence interval.

**Figure 3:**
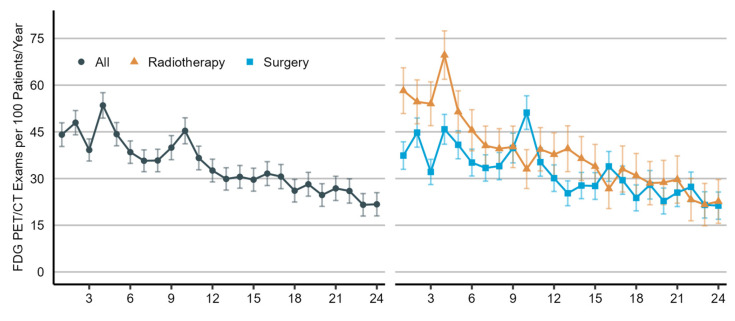
Utilization rate of [¹⁸F]FDG PET/CT imaging in the post-treatment management of non-small-cell lung cancer patients by month, which ranges from 1 to 24, with 0 representing the first 1-month interval of the two-year post-treatment period and 24 representing the last. Utilization rates are shown for all patients (on the **left**) and by type of initial treatment (on the **right**). Radiotherapy includes definitive chemoradiation or stereotactic body radiotherapy. The utilization rate is presented as the number of [¹⁸F]FDG PET/CT scans per 100 patients per year (predictive margins), accompanied by a 95% confidence interval.

As illustrated in Figure 2, the utilization rate of [¹⁸F]FDG PET/CT imaging increased considerably from 2007 to 2013 (predictive margin (pm), 10.4 [95% CI, 4.5 to 16.3] and 39.6 [95% CI, 36.5 to 42.7], respectively), but it has since remained stable. As shown in Figure 3, the utilization rate of [¹⁸F]FDG PET/CT imaging was highest in the fourth month of the two-year post-treatment period (pm, 54.1 [95% CI, 49.9 to 58.2]) and then declined for the remainder. During the first six months, the use of [¹⁸F]FDG PET/CT imaging was higher in patients who received curative-intent radiotherapy as the initial treatment than in patients who underwent surgical resection.

As presented in Table 2, the utilization rate of [¹⁸F]FDG PET/CT imaging was increased in patients who received curative-intent radiotherapy (+22.1% compared to surgical resection) and in patients with stage II–III disease (+14.2% and +20.0% compared to stage I, respectively). Moreover, the utilization rate was increased in 1-month intervals where post-treatment diagnostic procedures and interventions were performed, ranging from +33.8% for radiotherapy to +668.2% for “other diagnostic procedures” (ultrasound, MRI, endoscopy, or biopsy). Conversely, the utilization rate was decreased when systemic therapies were given. The utilization rate also varied greatly depending on the region, with a +111.6% higher utilization rate in the Region of Southern Denmark compared to the Region of Northern Denmark. As expected, the utilization rate was increased for patients who were enrolled in the [¹⁸F]FDG PET/CT arm of the SUPE_R trial.

## 4. Discussion

In this study, we aimed to investigate the trends and factors associated with the use of [¹⁸F]FDG PET/CT imaging in NSCLC patients after curative treatment. We analyzed data from a large cohort of 13,758 patients diagnosed with NSCLC between 2007 and 2020, representing the entire curatively treated NSCLC patient population in Denmark with adequate follow-up over that period. Our main findings include a significant increase in the use of [¹⁸F]FDG PET/CT after curative treatment from 2007 to 2013, followed by a period of stability. Patient characteristics associated with higher use of [¹⁸F]FDG PET/CT imaging included curative-intent radiotherapy as the initial curative treatment and stage II–III disease. In addition, we found that post-treatment interventions and diagnostic procedures were significant predictors of [¹⁸F]FDG PET/CT imaging use.

Our findings highlight the increasing role of [¹⁸F]FDG PET/CT imaging in the surveillance period after the curative treatment of NSCLC. A previous study on the use of [¹⁸F]FDG PET imaging after the curative treatment of NSCLC and colorectal cancer based on survival, epidemiology, and end results (SEERs)–Medicare data from the US found that the proportion of lung cancer patients who received FDG PET scans after curative treatment doubled from 2001 to 2009, from 11% to 25% [16]. In our study, we found that 32% of patients diagnosed in 2009–2010 received at least one [¹⁸F]FDG PET/CT scan, which is only slightly higher than what was found by Veenstra et al.

The increasing use of [¹⁸F]FDG PET/CT imaging after curative treatment could be attributed to several factors. First, a contributing factor may be the growing adoption of [¹⁸F]FDG PET/CT imaging for the preoperative staging of lung cancer and the availability of PET/CT systems. From 2007 to 2013, the proportion of patients staged with [¹⁸F]FDG PET/CT grew from 15% to 70%, and the number of PET/CT scanners in Denmark increased from 21 to 34 [17,18]. Secondly, there has been a growing emphasis on scheduled surveillance with CT after curative therapy, as supported by evidence and recommended by the European Society of Medical Oncology (ESMO) since 2010 [19]. From 2011, all patients who received curative treatment for NSCLC in Denmark were offered scheduled surveillance with CT every three months for the first two years after treatment and then every six months for a total of five years [20]. Consistent with this trend, our study found that the number of CT scans has grown by 17% annually during the study period, and the proportion of patients who received at least one CT during the two years after treatment increased from 77% in 2007–2008 to 97% in 2011–2012. The growing intensity of post-treatment surveillance might result in a higher number of positive or inconclusive findings, necessitating additional diagnostic tests, such as [¹⁸F]FDG PET/CT, to diagnose or rule out recurrent disease. In line with this reasoning, our analysis found that the use of [¹⁸F]FDG PET/CT imaging was strongly associated with the use of other diagnostic procedures, such as MRI, ultrasound, endoscopy, and biopsies.

In addition to the increasing use of [¹⁸F]FDG PET/CT imaging after curative therapy in general, we found that the use of [¹⁸F]FDG PET/CT imaging is higher in patients with stage II–III disease and in patients who received curative-intent radiotherapy as the initial curative therapy. These findings could indicate that the use of [¹⁸F]FDG PET/CT is increased when the risk of recurrence and post-radiation pneumonitis is elevated. Patients with stage II–III disease and those not eligible for surgical treatment have a high risk of recurrence during the first years following curative therapy compared to patients with early stage disease [1]. Moreover, patients who received radiotherapy are at risk of radiation-induced pneumonitis, which often occurs 1–6 months after treatment and can be difficult to distinguish from recurrence [21]. Prior studies have reported the benefits of [¹⁸F]FDG PET/CT imaging for distinguishing recurrent disease from benign, treatment-related changes detected in these patient populations [22,23]. As a result, the guidelines recommend using [¹⁸F]FDG PET/CT in this situation [9]. We observed that the frequency of [¹⁸F]FDG PET/CT imaging is highest during the first 12 months after curative therapy, especially after radiotherapy, when the risk of recurrence and radiation-induced pneumonitis is increased [24]. Finally, we found that all post-treatment interventions except systemic therapy were associated with the increased use of [¹⁸F]FDG PET/CT imaging. In this instance, [¹⁸F]FDG PET/CT imaging may be used to diagnose suspected recurrent disease for which treatment was initiated. However, it could also indicate that [¹⁸F]FDG PET/CT is performed for treatment planning or to assess the response to treatment.

It is important to consider the implications of increased resource utilization resulting from the noted increase in the use of [¹⁸F]FDG PET/CT imaging. The increased utilization of [¹⁸F]FDG PET/CT imaging undoubtedly carries an added economic cost. What is presently unclear—and should be explored further—is whether the health gains brought about by carrying out additional imaging outweigh or justify the additional healthcare resources, i.e., is such use of [¹⁸F]FDG PET/CT imaging cost-effective? Another consideration is the opportunity cost of increased [¹⁸F]FDG PET/CT imaging use. When operating in a health system characterized by scarce resources and funds (such as a public health system), utilizing resources for one purpose means they cannot be used for another. Therefore, this implies that the increase in [¹⁸F]FDG PET/CT imaging displaces other healthcare resources. This should also be further explored to establish that the utilization of healthcare resources is the most efficient.

One strength of this study is the long observation period, allowing for time trend identification. Another strength of this study is the reliability and comprehensiveness of the registry data from DLCR, which included virtually all patients in Denmark who received curative treatment for NSCLC during the study period. The comprehensiveness of the data is unique compared to other studies, such as the study from Veenstra et al., which was based on SEER–Medicare data, which only contains data on patients eligible for Medicare [16]. The data used in this analysis are also unique in that they explore the use of [¹⁸F]FDG PET/CT in a Danish healthcare system, where these imaging procedures carry zero cost to the patient, and the choice of imaging procedures is therefore unaffected by the ability to pay for healthcare. However, this is also a limitation of this study, as this may restrict the generalizability of our findings to other healthcare systems or patient populations. Another limitation is the retrospective design and the potential for residual confounding due to unobserved variables that limit our ability to make conclusions about the relationship between the use of [¹⁸F]FDG PET/CT imaging and the factors examined in our analysis. Moreover, the motivation for why each procedure was performed was unknown. For all imaging and treatment-related procedure codes, except surgical resection, the procedure code was not explicitly related to the diagnosis or treatment of lung cancer. However, a previous study on the use of [¹⁸F]FDG PET/CT in cancer in Denmark found that 63% of all [¹⁸F]FDG PET/CT scans were performed for the evaluation of cancer and that 26% of all [¹⁸F]FDG PET/CT scans performed were to evaluate lung cancer [25]. Therefore, it is reasonable to assume that most of the [¹⁸F]FDG PET/CT imaging performed during the first two years following lung cancer treatment is related to the post-treatment management of lung cancer. Nevertheless, post-treatment imaging and interventions could also be attributed to pre-existing conditions, treatment-related complications, or other diseases unrelated to the lung cancer diagnosis. As a result, assumptions based on the appropriateness of [¹⁸F]FDG PET/CT use in individual cases should be considered carefully.

## 5. Conclusions

[¹⁸F]FDG PET/CT is a valuable supplementary tool for managing patients after curative therapy for NSCLC, alongside scheduled surveillance with CT scans. The presented data suggest that there has been a substantial increase in the use of [¹⁸F]FDG PET/CT imaging for this application over the years. In agreement with the currently recommended uses of [¹⁸F]FDG PET/CT imaging after the curative therapy of NSCLC, our findings suggest that [¹⁸F]FDG PET/CT imaging plays a vital role in managing patients with stage II–III disease and those who received curative-intent radiotherapy who are at an increased risk of recurrence and radiation-induced pneumonitis. Further research is needed to establish the optimal use cases for [¹⁸F]FDG PET/CT imaging and to determine its impact on patient outcomes as well as healthcare system efficiency.

## Figures and Tables

**Table 1:** Patient characteristics and imaging by year of diagnosis.

Characteristics	Total	2007–2008	2009–2010	2011–2012	2013–2014	2015–2016	2017–2018	2019–2020
Number of patients	13758	1180	1430	1823	2021	2333	2589	2380
Age in years–mean (SD)	68.2 (8.9)	66.1 (9.1)	66.2 (8.9)	67.1 (9)	68.2 (9.1)	68.5 (8.8)	69 (8.5)	70 (8.4)
Stage ^1^–N (%)								
I	7595 (55)	678 (57)	746 (52)	967 (53)	1089 (54)	1244 (53)	1446 (56)	1425 (60)
II	2774 (20)	206 (17)	317 (22)	364 (20)	424 (21)	482 (21)	526 (20)	455 (19)
III	3389 (25)	296 (25)	367 (26)	494 (27)	508 (25)	607 (26)	617 (24)	500 (21)
Initial treatment–N (%)								
Surgical resection	9401 (68)	935 (79)	1067 (75)	1232 (68)	1312 (65)	1503 (64)	1738 (67)	1614 (68)
Radiotherapy ^2^	4357 (32)	245 (21)	363 (25)	593 (32)	709 (35)	830 (36)	851 (33)	766 (32)
Region–N (%)								
Northern Denmark	1733 (13)	145 (12)	187 (13)	212 (12)	247 (12)	315 (14)	355 (14)	272 (11)
Central Denmark	3070 (22)	277 (23)	301 (21)	359 (20)	445 (22)	520 (22)	597 (23)	571 (24)
Southern Denmark	3467 (25)	315 (27)	320 (22)	459 (25)	508 (25)	604 (26)	700 (27)	561 (24)
Capital Region	3440 (25)	309 (26)	425 (30)	483 (26)	526 (26)	521 (22)	565 (22)	611 (26)
Zealand	2048 (15)	134 (11)	197 (14)	312 (17)	295 (15)	373 (16)	372 (14)	365 (15)
Follow-up imaging modalities–N (%)								
CT	13134 (95)	908 (77)	1283 (90)	1778 (97)	1990 (98)	2299 (99)	2547 (98)	2329 (98)
FDG PET/CT	5327 (39)	274 (23)	453 (32)	781 (43)	864 (43)	949 (41)	1104 (43)	902 (38)

^1^ Clinical stage or pathological stage if available; ^2^ definitive chemoradiation or stereotactic body radiotherapy. FDG PET/CT, [¹⁸F]fluorodeoxyglucose positron emission tomography/computed tomography; CT, computed tomography.

**Table 2:** Frequency of ^18^F-FDG PET/CT imaging (predictive margins).

Predictor	Frequency of FDG PET/ CT Imaging (95% CI)	Difference (95% CI), Percentage Change	*p* Value
Age–years (at representative values)			
60	37.2 (36.2–38.3)	Reference	
70	35.5 (34.7–36.3)	−4.6 (−7.1–−2)	<0.001
80	33.9 (32.6–35.2)	−9 (−13.8–−4)	<0.001
Stage ^1^			
I	33.2 (32.1–34.3)	Reference	
II	37.9 (36.1–39.8)	14.2 (7.4–21.5)	<0.001
III	39.8 (38.1–41.6)	20 (12.9–27.5)	<0.001
Initial treatment			
Surgical resection	33.5 (32.5–34.5)	Reference	
Radiotherapy ^2^	40.9 (39.3–42.5)	22.1 (15.7–28.9)	<0.001
Region			
Northern Denmark	22.3 (20.5–24)	Reference	
Central Denmark	29.1 (27.6–30.7)	30.8 (18.9–43.9)	<0.001
Southern Denmark	47.1 (45.3–48.9)	111.6 (93.3–131.7)	<0.001
Capital Region	39.5 (37.8–41.1)	77.2 (61.3–94.6)	<0.001
Zealand	32.5 (30.6–34.4)	45.9 (31.1–62.3)	<0.001
Inclusion in the SUPE_R study			
Not Included	34.5 (33.7–35.3)	Reference	
FDG PET/CT arm	154 (137.8–170.2)	346.1 (302.2–394.9)	<0.001
Control arm	27.8 (21.4–34.2)	−19.6 (−35.6–0.5)	0.055
Procedures ^3^			
Surgical resection	65.2 (44.9–85.4)	81.6 (25.2–163.5)	0.002
Radiotherapy ^4^	47.7 (41.9–53.5)	33.8 (16.9–53.1)	<0.001
Systemic therapy ^5^	31 (28.5–33.4)	−14.9 (−23–-6)	0.002
Diagnostic procedures ^6^	199.9 (191.9–207.9)	668.2 (627.4–711.2)	<0.001

^1^ Clinical stage or pathological stage if available; ^2^ definitive chemoradiation or stereotactic body radiotherapy; ^3^ procedures performed during the two-year follow-up period after the initial curative therapy; ^4^ any type of external beam radiotherapy; ^5^ any type of chemotherapy, targeted therapy, or immune checkpoint inhibitor; ^6^ endoscopy, biopsy, ultrasound, or magnetic resonance imaging. FDG PET/CT, [¹⁸F]fluorodeoxyglucose positron emission tomography/computed tomography.

## Data Availability

The datasets presented in this article are not readily available due to the nature of the data, which includes personal identifiable information. Requests to access the datasets should be directed to The Danish Clinical Quality Program–National Clinical Registries (RKKP).

## Supplementary Materials

The following supporting information can be downloaded at: https://www.mdpi.com/article/10.3390/diagnostics14020233/s1, Table S1: DMCCS Codes.
